# Diagnostic and predictive values of serum metabolic profiles in sudden sensorineural hearing loss patients

**DOI:** 10.3389/fmolb.2022.982561

**Published:** 2022-09-06

**Authors:** Xiangsheng Wang, Yan Gao, Ruirui Jiang

**Affiliations:** ^1^ Department of Otolaryngology-Head and Neck Surgery, Urumqi Maternal and Child Health Care Hospital, Urumqi, China; ^2^ Department of Otolaryngology-Head and Neck Surgery, The Second Affiliated Hospital of Xin Jiang Medical University, Urumqi, China; ^3^ Department of Pharmacy, The First People’s Hospital of Urumqi (Children’s Hospital), Urumqi, China

**Keywords:** sudden sensorineural hearing loss, hearing recovery, metabolomics, metabolites, biomarker

## Abstract

Sudden sensorineural hearing loss (SSNHL) is an otologic emergency, and metabolic disturbance is involved in its pathogenesis. This study recruited 20 SSNHL patients and 20 healthy controls (HCs) and collected their serum samples. Serum metabolites were detected by liquid chromatography-mass spectrometry, and metabolic profiles were analyzed. All patients were followed up for 3 months and categorized into recovery and non-recovery groups. The distinctive metabolites were assessed between two groups, and their predictive values for hearing recovery were evaluated. Analysis results revealed that SSNHL patients exhibited significantly characteristic metabolite signatures compared to HCs. The top 10 differential metabolites were further analyzed, and most of them showed potential diagnostic values based on receiver operator characteristic (ROC) curves. Finally, 14 SSNHL patients were divided into the recovery group, and six patients were included in the non-recovery group. Twelve distinctive metabolites were observed between the two groups, and ROC curves demonstrated that N4-acetylcytidine, p-phenylenediamine, sphingosine, glycero-3-phosphocholine, and nonadecanoic acid presented good predictabilities in the hearing recovery. Multivariate analysis results demonstrated that serum N4-Acetylcytidine, sphingosine and nonadecanoic acid levels were associated with hearing recovery in SSNHL patients. Our results identified that SSNHL patients exhibited distinctive serum metabolomics signatures, and several serum biomarkers were proved to be potential in predicting hearing recovery. The discriminative metabolites might contribute to illustrating the mechanisms of SSNHL and provide possible clues for its treatments.

## Introduction

Sudden sensorineural hearing loss (SSNHL) is an otologic emergency which is defined as a sharp hearing loss of ≥30 dB in three sequential frequencies within 72 h (Huang et al., 2022a; Huang et al., 2022b). Previous epidemiological investigations showed that the incidence of SSNHL ranged from 5 to 20 per 100,000 individuals (Chen et al., 2021; Doweck et al., 2022). Currently, the pathogenic causes of SSNHL are still debated, and several possible etiologies have been proposed, including viral infection, vascular disorders, trauma and autoimmune diseases (Shen et al., 2021; Wu et al., 2022a; Feng et al., 2022). As a medical emergence, detailed examination to determine possible causes and early treatment onset is extremely important to improve hearing prognosis in SSNHL patients (Jiang et al., 2021; Huo et al., 2022). Although numerous studies were performed to determine the diagnostic and prognostic biomarkers in SSNHL, no reliable indicator was available in practice (Huo et al., 2022; Park et al., 2022). Recently, an increasing number of evidence showed that metabolic diseases were independent risk factors for the occurrence of SSNHL, and pathoglycemia and dyslipidemia significantly affected hearing recovery (Doo et al., 2020; Chen et al., 2022a; Simões et al., 2022). Shen et al. (2021) reported that glycosylated hemoglobin A1c levels were increased in SSNHL patients and associated with disease severity and audiogram types. Zhang et al. (2019) found that metabolic syndrome negatively affected the prognosis of SSNHL, and the hearing recovery was poorer with an increase in the number of metabolic syndrome components. In another study, serum lipids were proven to be correlated with the incidence and prognosis of SSNHL (Li et al., 2021). Although the metabolic disturbances of carbohydrates and lipids were demonstrated to act crucial roles in SSNHL, the serum metabolic profiles in SSNHL patients were undefined.

Metabolomics is a burgeoning branch of omics science which is performed in a biological system to capture metabolic perturbations (Chen et al., 2022b; Roverso et al., 2022). Metabolomic is a systems biology approach that attempts to explore pathogenesis and diagnostic markers of clinical diseases, including malignant tumours, cardiovascular and inflammatory diseases, and plentiful diagnosis and prognosis assessment models were constructed based on novel metabolites and metabolic pathways which contributed to optimizing disease diagnosis and surveillance and fostering the development of precise treatment (Guo et al., 2022; Sawicka et al., 2022). Recently, a growing number of otologists paid close attention to metabolomics and its application prospect in inner ear diseases, and they conducted metabolomic protocols on plasma, urine, perilymph samples and *in vitro* cells derived from hearing loss patients and animal models and identified various disease-specific metabolic pathways and metabolites, these results suggested that metabolomics was a reliable method to clarify inner ear pathologies in hearing loss (Ji et al., 2019; Gaboon et al., 2020; Boullaud et al., 2022; Kaderbay et al., 2022). Pirttilä and others applied untargeted metabolomics protocol in cochlear perilymph of the inner ear of noise-exposed Guinea pigs and observed a clear difference in the perilymph metabolic fingerprint (Pirttilä et al., 2019). A recent study reported that the plasma metabolomic profiling in workers with noise-induced hearing loss was significantly changed compared to healthy volunteers, and 12 plasma metabolites and seven metabolic pathways were identified to be disturbed (Miao et al., 2021). Although metabolomics studies were successfully utilized in several inner diseases, its application in SSNHL was extremely limited. Up to now, only a preliminary study with a small sample size assessed the metabolomics in the urine samples of 5HCs and 13 SSNHL patients and identified several distinctive metabolites, but the predictive values of these biomarkers in hearing recovery were not evaluated (Carta et al., 2017). Besides, no prior study has evaluated metabolites and metabolic pathway changes in the serum of SSNHL patients and explored objective biomarkers for predicting the hearing prognosis before treatment onset based on metabolomics.

Therefore, we aimed to discover serum metabolic signatures of SSNHL patients and evaluate the capacities of distinctive metabolites in predicting the prognosis of SSNHL in the present study.

## Materials and methods

### Patients and settings

In this study, a total of 20 consecutive patients with SSNHL and 20 gender- and age-matched HCs were enrolled between June 2021 and October 2021 in our department. All included subjects signed informed consent, and the present study was approved by the ethical committee in our hospital (No.20191209-01, December 9^th^, 2019). SSNHL was diagnosed by pure tone audiometry (PTA), referring to the guidelines provided by the Chinese Medical Association of Otolaryngology (CMAO) (Editorial Board of Chinese Journal of Otorhinolaryngology H, 2015). All patients underwent detailed routine examinations and laboratory tests. These patients were excluded: refused to participate in the study; with a determined aetiology; treatment delay time >30 days; age <18 years or >65 years; antidiabetics or hypolipidemic drugs consumption within a month; severe heart, kidney, or other metabolic diseases; accompanying any acute inflammation; incomplete clinical records or follow-up data. The demographic and clinical data before the onset of treatments were collected, including age, gender, body mass index (BMI, weight in kilograms divided by the square of height in meters), side, accompanied symptoms and audiogram type. The audiogram was divided into ascending, descending, flat and profound, as previously described (Xie et al., 2020a; Kim et al., 2020).

### Treatment and hearing outcome evaluation

All SSNHL patients received PTA before the onset of treatment, and they were treated with standard treatment protocol in our department, including oral or intravenous steroids, and adjuvant blood flowing promoting agents as our previous publication described (Wang et al., 2019). PTA was conducted before the first day of treatment onset, and the initial hearing threshold was calculated based on the arithmetic mean of thresholds at 500, 1000, 2000, and 4000 Hz. During the treatment, PTA was performed every 2–3 days to monitor the post-treatment audiological changes and make decisions to terminate treatment. All patients were followed for more than 3 months, and final hearing improvements were defined based on the hearing improvement between initial and 3 months post-treatment hearing thresholds. Hearing recovery was categorized into complete recovery, marked recovery, slight recovery, and no recovery referring to Siegel’s criteria, and complete recovery and marked recovery were regarded as recovery, and slight recovery and no recovery were recognized as non-recovery as most previous studies described (Siegel, 1975; Xiong et al., 2022).

### Serum sample collection and preparation

Five mL of fasting peripheral blood from SSNHL patients before treatment and HCs were collected and stored for 1 h at room temperature. All collected samples were isolated and centrifuged at 3500 rpm at room temperature for 15 min, and supernatants were harvested and stored at −80°C until use. A 100 μL test specimen was mixed with 300 uL methanol containing internal standard for the preparation for LC-MS analysis. The mixture was further vortexed for 30 s and sonicated for 10 min, then centrifuged at 12,000 rpm at 4°C for 20 min. The liquid supernatant was collected and set to a second centrifugation at 13,000 rpm at 4°C for 20 min. The supernatants were transferred to a fresh glass vial for metabolomics analysis. A quality control (QC) sample was prepared by mixing an equal aliquot of the supernatants derived from test specimens to evaluate the reproducibility and reliability of the metabolomics analytical system (Xie et al., 2021; Zeng et al., 2022).

### LC-MS analysis

Metabonomic profiles of included samples were detected with 290 Infinity series ultrahigh-performance liquid chromatography (UHPLC) System (Waters Corporation, Milford, MA, United States), equipped with a UPLC BEH Amide column (2.1 mm×100 mm, 1.7 μm). Ultrahigh-performance liquid chromatography (UHPLC) (Waters, Milford, United States) system equipped with AB SCIEX Triple TOF 5600 System (AB SCIEX, Framingham, United States). The mobile phase consisted of 25 mmol/L ammonium acetate and 25 mmol/L ammonia hydroxide in water (A) and acetonitrile (B). All analyzed samples were kept at 4°C, and the temperature of the column was kept at 25°C. The analysis was performed with an elution gradient as previously described (Darwish et al., 2021; Wu et al., 2022b). The Triple TOF 6600 mass spectrometry (AB Sciex, Boston, MA, United States) was applied to catch MS/MS spectra on an information-dependent basis (IDA) during the LC/MS experiment.

### Data processing and analysis

The original data were converted to the mzXML format with Proteo Wizard and analyzed by the R package version. These processes include baseline removing, peak identification, peak alignment and integration, and retention time adjustment (Chen et al., 2022b; Hansen et al., 2022). Metabolites identification was conducted based on the In-house MS2 database. The resultant data was exported to SIMCA (Version 14.1, Umetrics, Umea, Sweden) for sequential analysis. Principal component analysis (PCA) and orthogonal partial least square-discriminant analysis (OPLS-DA) models were used to explore the metabolic difference between the groups. Variable importance for project (VIP) values were applied to evaluate the metabolite’s contribution, and metabolites with VIP >1.0 and *p*-values <0.05 were regarded as potential biomarkers (Xie et al., 2020b). Volcano plot and heat map were generated to exhibit the metabolic regulations of the remarkable changes between different groups (Wu et al., 2022b; Roverso et al., 2022). To determine diagnostic and predictive values of distinctive metabolites, receiver operating characteristics (ROC) curves were performed, and the area under the curve (AUC) was calculated to evaluate the predictive abilities.

### Statistical analysis

Numerical variables with normal distribution were shown as mean ± standard deviation (SD), and those without normal distribution were described as the median and interquartile range (IQR). Categorical variables are presented as numbers and percentages. Student’s t-test or Mann-Whitney U test was utilized to compare the differences between continuous variables, and the Chi-square test or Fisher’s exact test was used for categorical variables. Multivariate analysis was performed to assess the association between selected metabolite levels and auditory recovery. All statistical analyses were conducted with SPSS statistics software version23.0 (IBM, Chicago, IL, United States), and *p* values <0.05 were defined as statistically significant.

## Results

### Baseline characteristics of all participants

A total of 20 SSNHL patients and 20 HCs were included in this study. The demographic and clinical characteristics of all subjects were displayed in Table 1. No significant difference was observed in terms of age, gender and BMI between SSNHL and HC groups (*p* > 0.05).

**TABLE 1:** Demographic and clinical parameters of HC and SSNHL patients.

Parameters	HC (*n* = 20)	SSNHL (*n* = 20)	*p* value
Age, year	37.0 (32.3, 42.8)	41.5 (30.0, 44.8)	0.605
Male/female	12/8	10/10	0.751
BMI, kg/m^2^	23.1 (21.1, 24.3)	22.0 (21.1, 25.0)	0.284
Side, left/right		11/9	
Tinnitus, n (%)		15 (75.0)	
Vertigo, n (%)		9 (45.0)	
Initial hearing thresholds, dB		62.5 (35.0, 72.5)	
Audiogram type, n (%)			
Ascending		6 (30.0)	
Descending		4 (20.0)	
Flat		5 (25.0)	
Profound		5 (25.0)	
Recovery, n (%)			
Complete recovery		6 (30.0)	
Marked recovery		8 (40.0)	
Slight recovery		4 (20.0)	
No recovery		2 (10.0)	

HC, healthy control; SSNHL, sudden sensorineural hearing loss; BMI, body mass index.

### Distinct clustering of metabolites for SSNHL group vs. HC group

**FIGURE 1:**
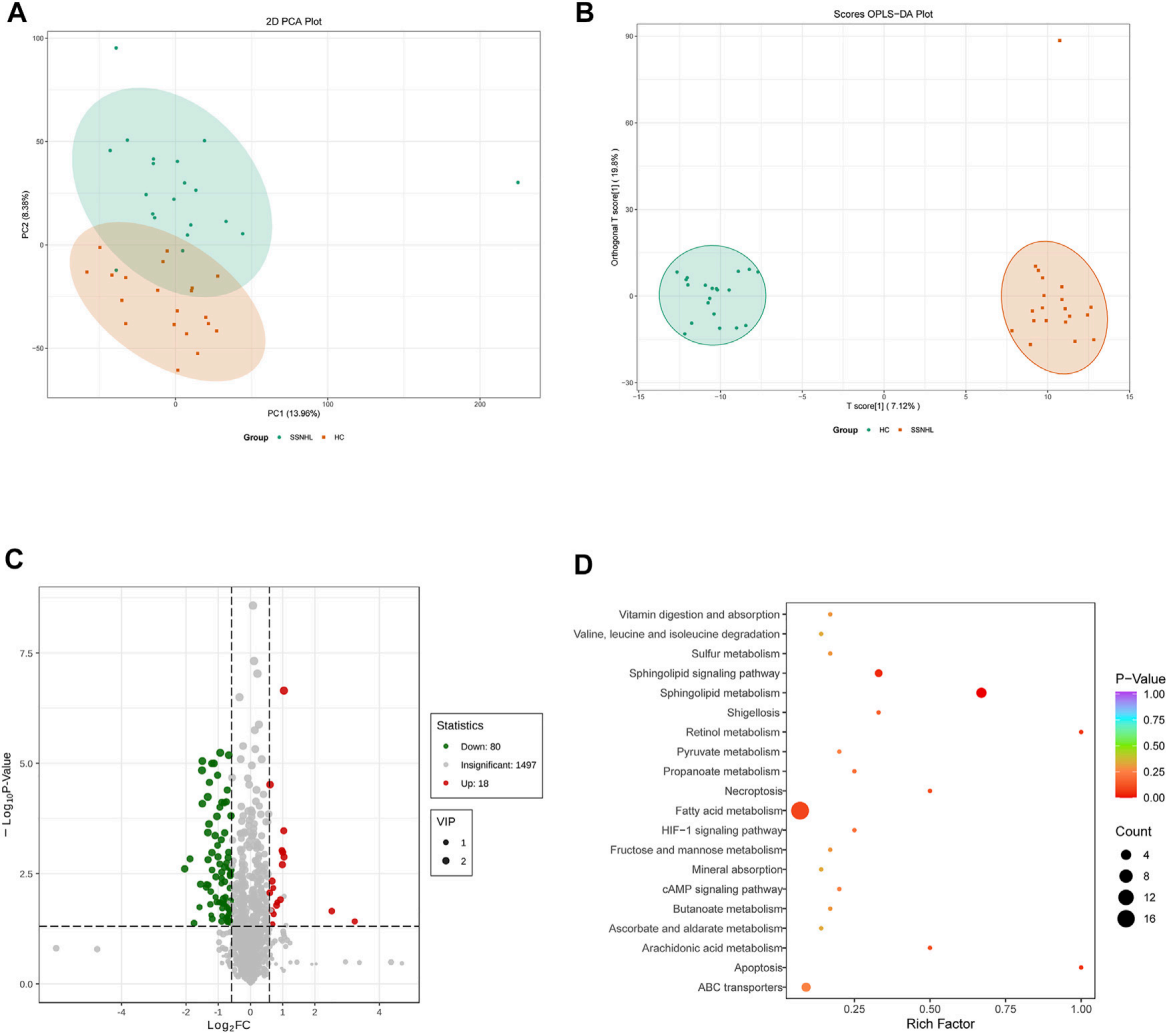
Serum metabolomics analysis of samples from SSNHL and HC groups. **(A)** PCA model and **(B)** OPLS-DA showed significant discrimination of SSNHL patients from HCs based on serum metabolomic profile; **(C)** volcano plot of differentially expressed metabolites; **(D)** bubble plot of enriched metabolic pathways. SSNHL, sudden sensorineural hearing loss; HC, healthy control; PCA, principal component analysis; OPLS-DA, orthogonal partial least square-discriminant analysis.

**FIGURE 2:**
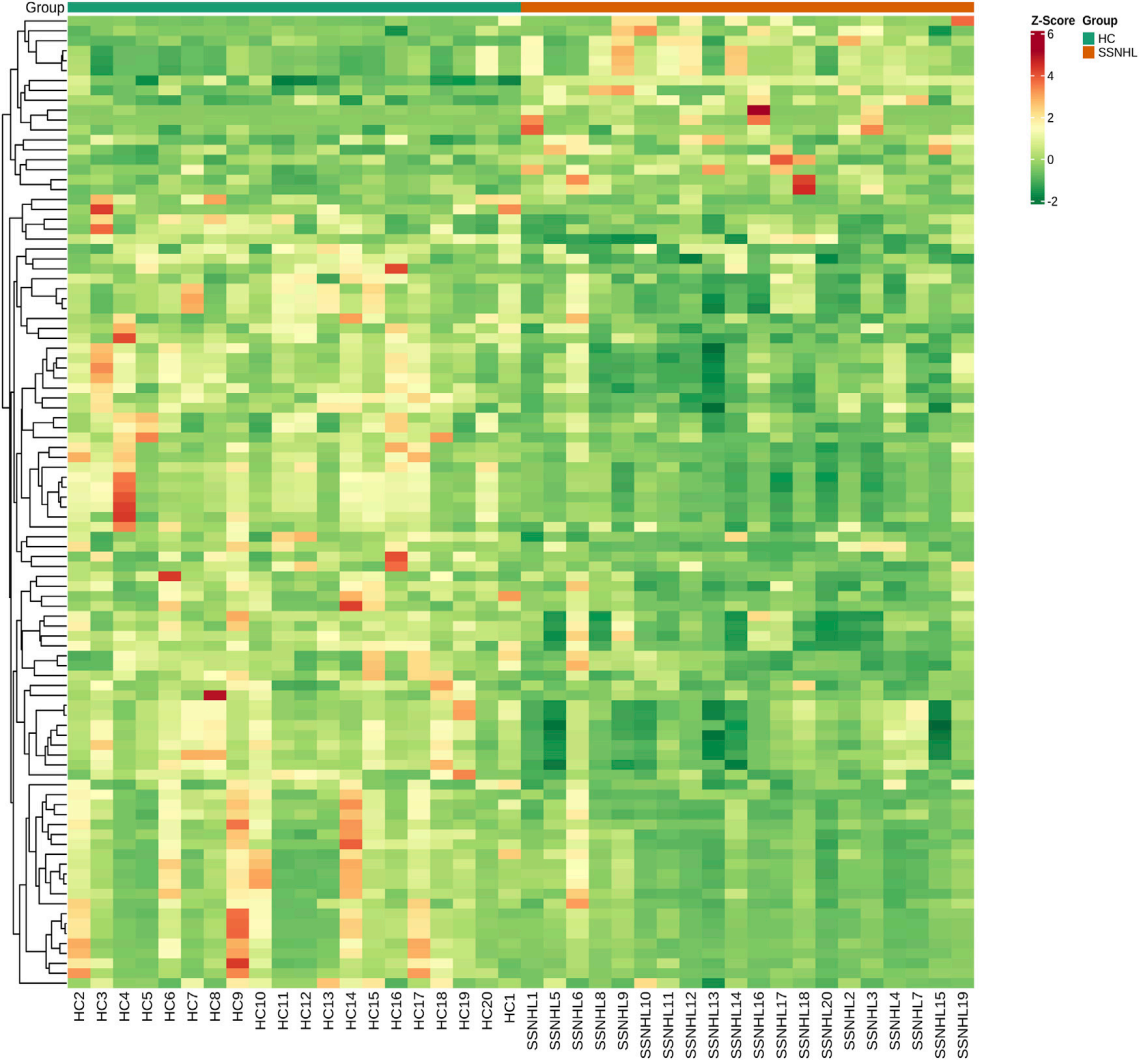
Heat map of the 98 discriminative metabolites between SSNHL group and HC group. SSNHL, sudden sensorineural hearing loss; HC, healthy control.

**FIGURE 3:**
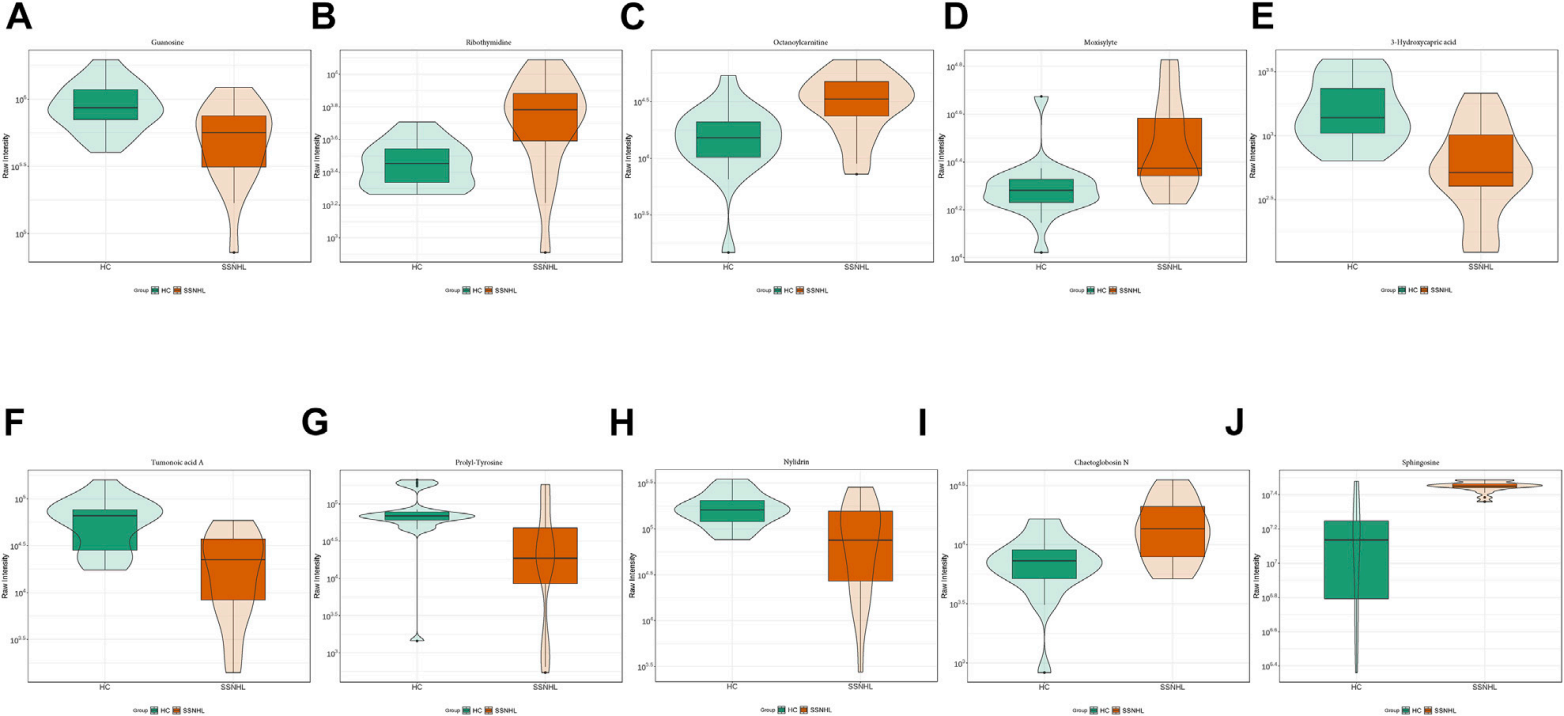
Top 10 most discriminant metabolites **(A–J)** in their relative levels between SSNHL group and HC group. SSNHL, sudden sensorineural hearing loss; HC, healthy control.

**FIGURE 4:**
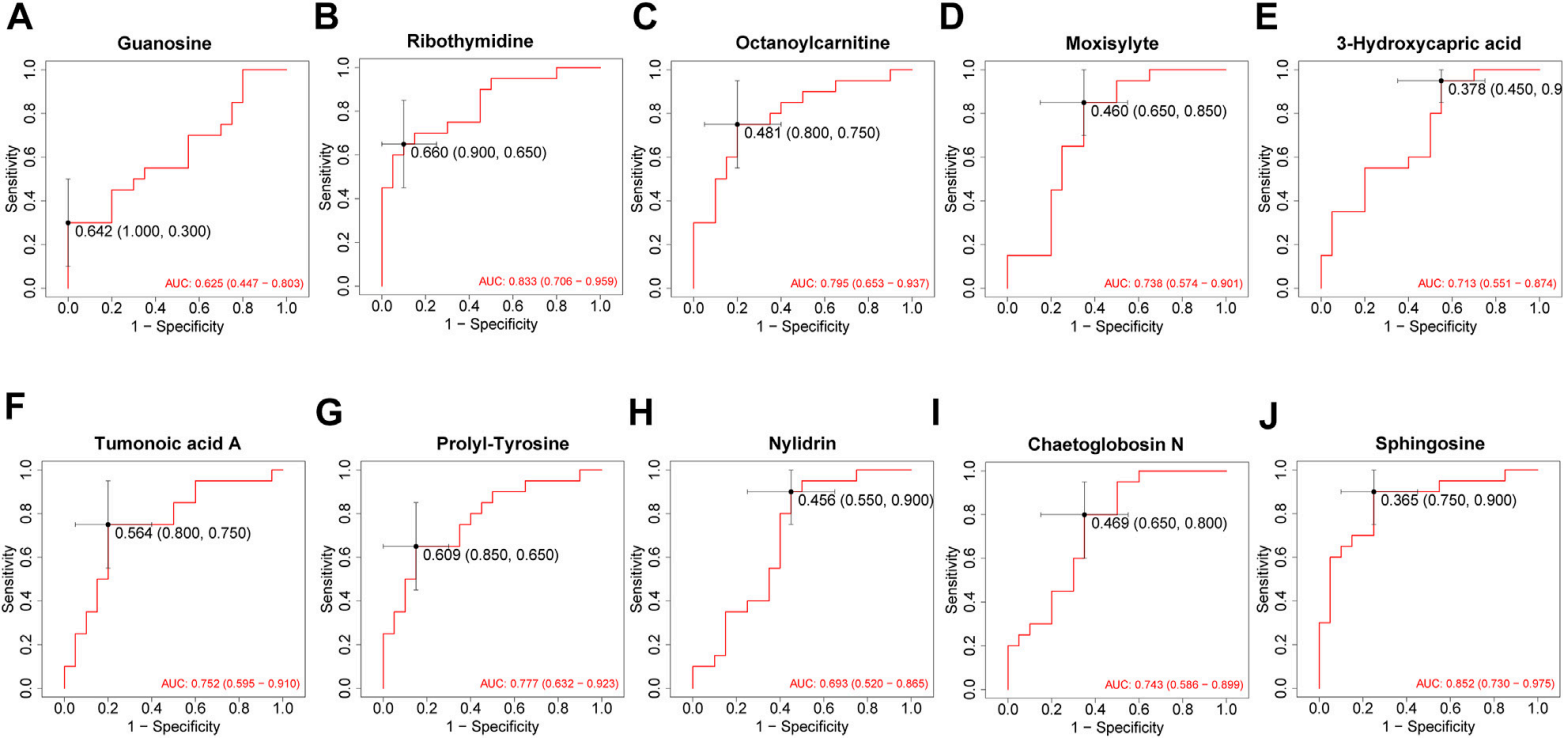
ROC analysis of top 10 most discriminant metabolites **(A–J)** for distinguishing SSNHL patients and HCs. ROC: receiver operating characteristics; SSNHL, sudden sensorineural hearing loss; HC, healthy control.

A total of 1943 metabolites were obtained in tested samples, and 1595 of them were identified as known substances. Based on these metabolites, PCA and OPLS-DA models were constructed, and the results presented clear and distinctive clustering between the serum of SSNHL patients and HCs (Figures 1A,B), suggesting SSNHL patients exhibited discriminative metabolic profiles in comparison with HCs. The volcano plot in Figure 1C showed that 98 metabolites were changed, including 18 up-regulated and 80 down-regulated between SSNHL and HC groups, and the most important disturbed pathways were fatty acid metabolism and sphingolipid metabolism (Figure 1D). The differential metabolites could be divided visually in the heat map (Figure 2). The top 10 differential metabolites (guanosine, ribothymidine, octanoylcarnitine, moxisylyte, 3-hydroxycapric acid, tumonoic acid A, Prolyl-tyrosine, nylidrin, chaetoglobosin N, and sphingosine) were listed in Table 2 referring to the contribution of VIP, and their relative levels in the serum were compared in Figure 3. ROC curves were constructed to evaluate the performance of the above 10 remarkable metabolites and the results showed that serum ribothymidine and sphingosine exhibited promising diagnostic values (Figure 4).

**TABLE 2:** Top ten distinctive metabolites between SSNHL patients and HCs.

Metabolites	VIP	FC	Regulation	*p*-value	Classification of metabolites
Guanosine	2.56	0.355	down	<0.001	Nucleotide and its metabolomics
Ribothymidine	2.44	2.523	up	<0.001	Pyrimidine nucleosides
Octanoylcarnitine	2.387	1.987	Up	0.002	Fatty Acyls
Moxisylyte	2.381	2.265	Up	0.001	Prenol lipids
3-Hydroxycapric acid	2.322	0.341	down	0.005	Hydroxy acids and derivatives
Tumonoic acid A	2.266	0.355	down	<0.001	Carboxylic acids and derivatives
Prolyl-tyrosine	1.978	0.410	down	<0.001	Carboxylic acids and derivatives
Nylidrin	1.913	0.439	down	0.004	Benzene and its derivatives
Chaetoglobosin N	1.782	1.672	Up	0.001	Cytochalasans
Sphingosine	1.71	2.756	Up	0.006	Sphingolipids

SSNHL, sudden sensorineural hearing loss; HC, healthy control; VIP, variable importance for project; FC, fold change.

### Metabolomic signatures for predicting hearing recovery

**FIGURE 5:**
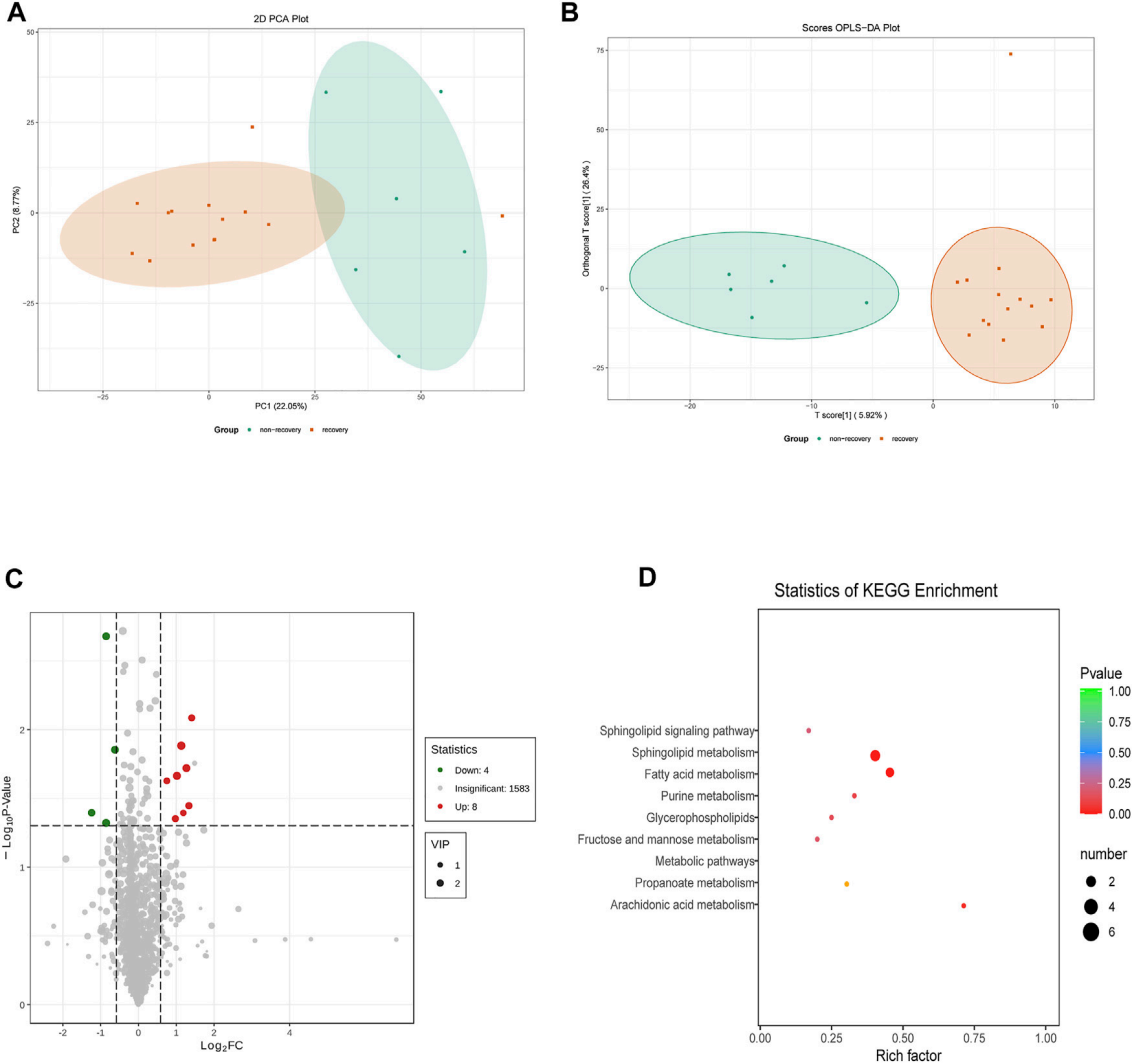
Serum metabolomics analysis of samples from recovery group and non-recovery group. **(A)** PCA model and **(B)** OPLS-DA showing significantly discriminative serum metabolomic profile between recovery and non-recovery groups; **(C)** volcano plot of differentially expressed metabolites; **(D)** bubble plot of enriched metabolic pathways. PCA, principal component analysis; OPLS-DA, orthogonal partial least square-discriminant analysis.

**FIGURE 6:**
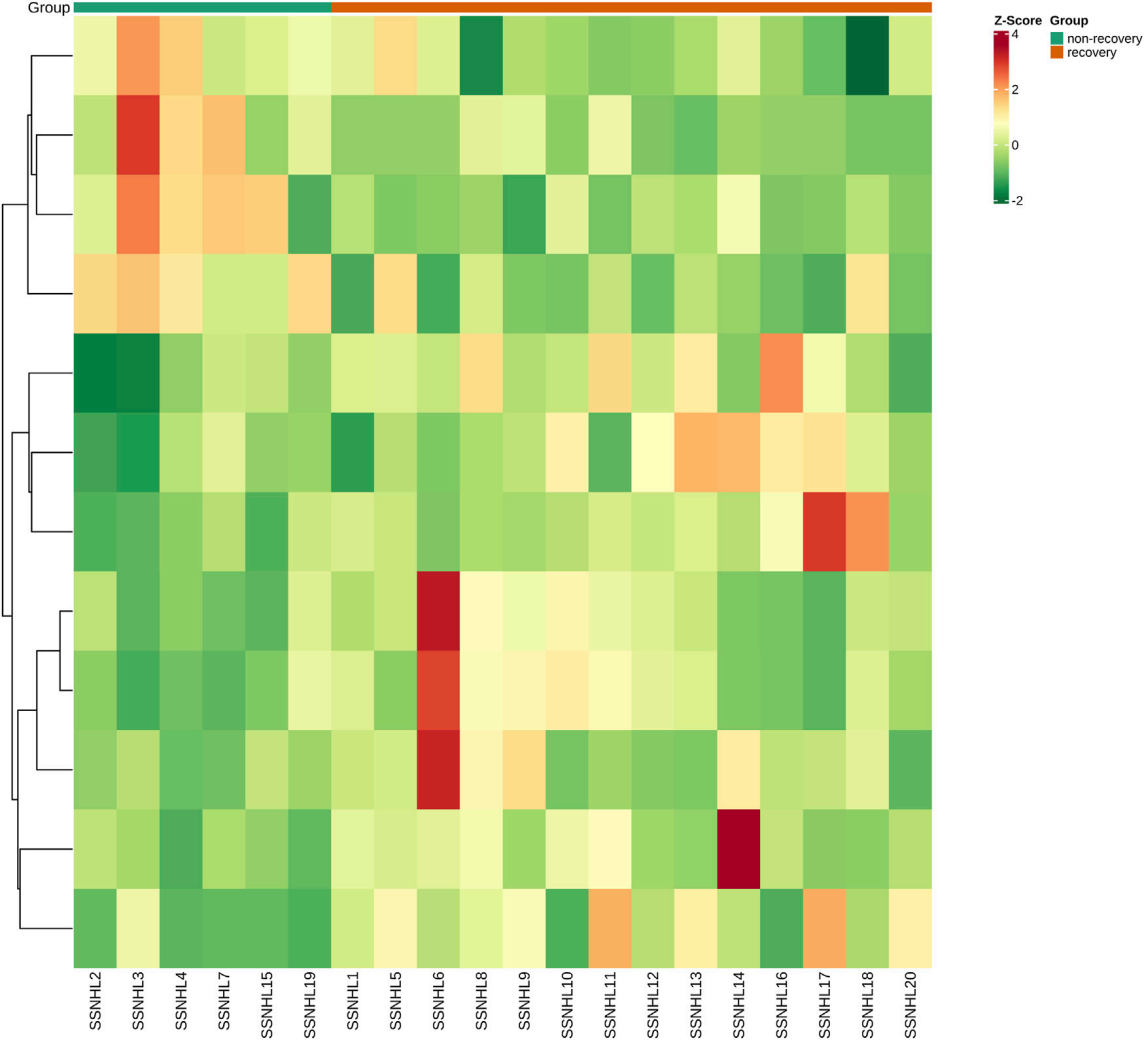
Heat map of the 12 discriminative metabolites between recovery and non-recovery groups.

**FIGURE 7:**
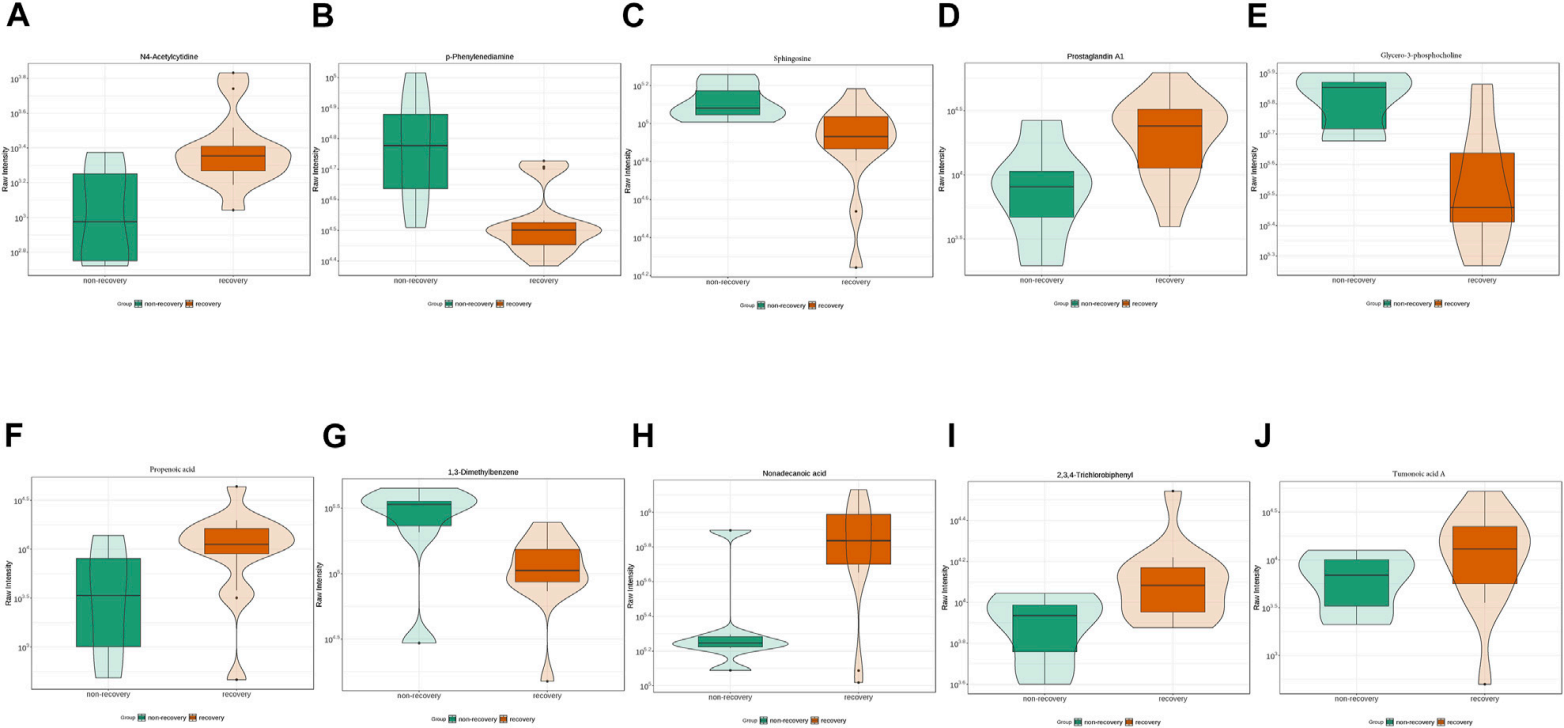
Top 10 most discriminant metabolites **(A–J)** in their relative levels between recovery and non-recovery groups.

**FIGURE 8:**
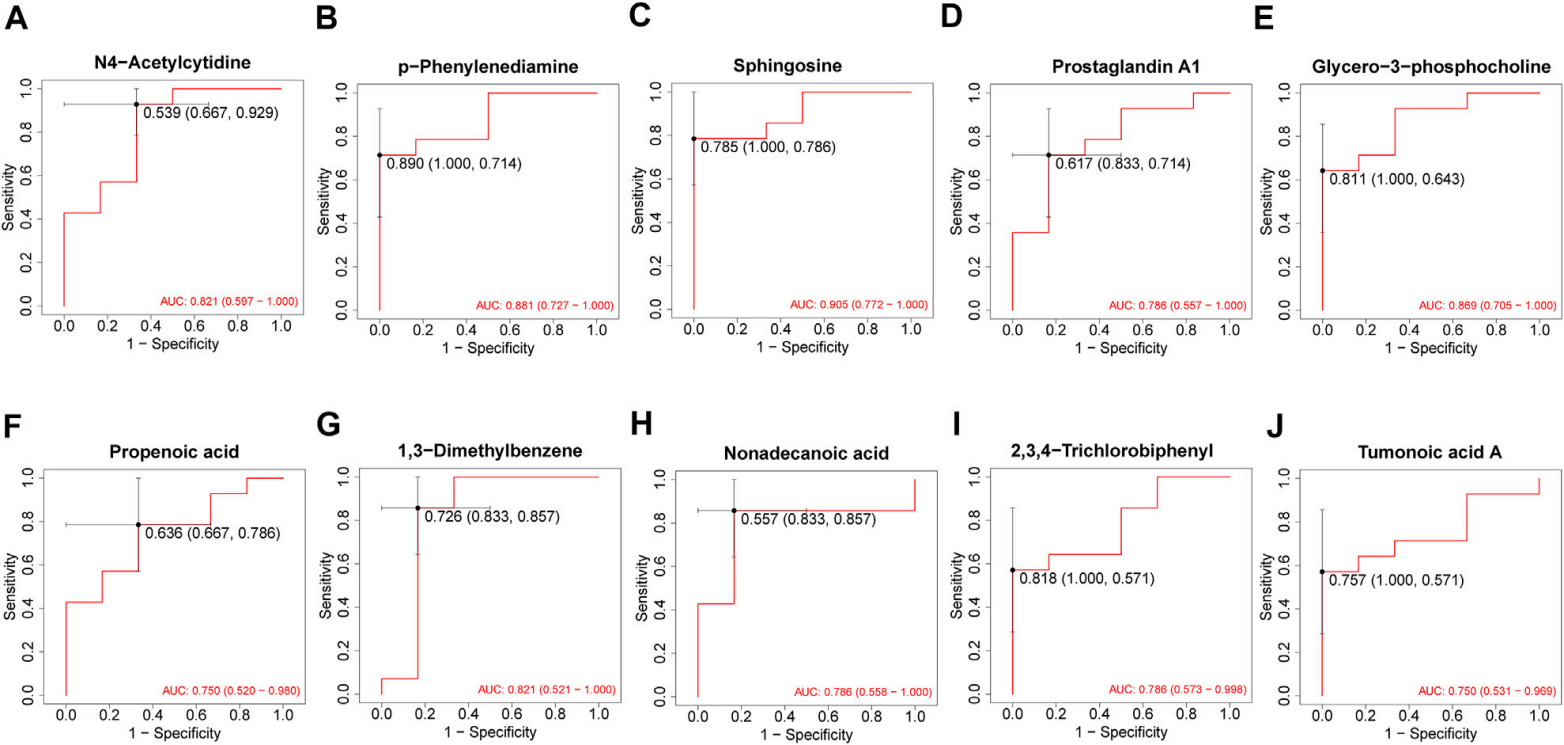
ROC analysis of top 10 most discriminant metabolites **(A–J)** for predicting a hearing recovery in SSNHL patients. ROC: receiver operating characteristics; SSNHL, sudden sensorineural hearing loss.

After 3 months of follow-ups, 14 patients were included in the recovery, and six patients were categorized into the non-recovery group. Table 3 exhibited the demographic and clinical characteristics of patients between the two groups. The post-treatment hearing thresholds were lower, and hearing improvement was greater in the recovery group than non-recovery group (*p* < 0.05), but no statistical difference was found between the two groups in age, gender, BMI, side, tinnitus, vertigo, initial hearing thresholds and audiogram type (*p* > 0.05). PCA and OPLS-DA models in Figures 5A,B presented distinctive serum metabolomics profiles between recovery and non-recovery groups, and 12 metabolites were disturbed, including eight up-regulated and four down-regulated between two groups which involving fatty acid metabolism and sphingolipid metabolism pathways (Figures 5C,D). The heat map in Figure 6 exhibited a visually discriminative metabolic fingerprint between recovery and non-recovery groups. Results of the top 10 most discriminant metabolites were shown in Table 4, and their relative levels were comparatively shown in Figure 7. The potential metabolites between two groups with good predictabilities (AUC>0.8) were N4-acetylcytidine, p-phenylenediamine, sphingosine, glycero-3-phosphocholine and nonadecanoic acid (Figure 8). Moreover, both adjusted and unadjusted logistic regression analysis results suggested that multivariate analysis results demonstrated that serum N4-Acetylcytidine, sphingosine, and nonadecanoic acid levels were associated with hearing recovery in SSNHL patients (Table 5).

**TABLE 3:** Demographic and clinical parameters between recovery and non-recovery groups.

Parameters	Recovery (*n* = 14)	Non-recovery (*n* = 6)	*p*-value
Age, year	38.5 (38.0, 44.3)	42.0 (34.0, 48.0)	0.438
Male/female	6/8	4/2	0.628
BMI, kg/m^2^	21.7 (21.1, 25.1)	21.1 (21.4, 23.6)	0.340
Side, left/right	8/6	3/3	1.000
Tinnitus, n (%)	12 (85.7)	3 (50.0)	0.131
Vertigo, n (%)	7 (50.0)	2 (33.3)	0.642
Initial hearing thresholds, dB	61.3 (35.0, 70.0)	65.0 (42.5, 75.0)	0.867
Post-treatment hearing thresholds, dB	32.5 (27.5, 40.0)	57.5 (41.3, 67.5)	0.006
Hearing improvement, dB	30.0 (22.5, 37.5)	6.3 (5.0, 10.0)	<0.001
Audiogram type, n (%)			0.172
Ascending	6 (42.9)	0 (0)	
Descending	3 (21.4)	1 (16.7)	
Flat	3 (21.4)	2 (33.3)	
Profound	2 (14.3)	3 (50.0)	

BMI, body mass index.

**TABLE 4:** Top ten distinctive metabolites between recovery and non-recovery groups.

Metabolites	VIP	FC	Regulation	*p*-value	Classification of metabolites
N4-Acetylcytidine	2.823	2.19	Up	0.013	Pyrimidine nucleosides
p-Phenylenediamine	2.651	0.552	down	0.005	Benzene and its derivatives
Sphingosine	2.613	2.024	up	0.021	Sphingolipids
Prostaglandin A1	2.514	2.408	up	0.019	Fatty acyls
Glycero-3-phosphocholine	2.419	0.649	down	0.014	Glycerophospholipids
Propenoic acid	2.401	2.076	up	0.002	Fatty Acyls
1,3-Dimethylbenzene	2.187	0.423	down	0.040	Benzene and its derivatives
Nonadecanoic acid	2.023	2.521	up	0.036	Fatty acyls
2,3,4-Trichlorobiphenyl	1.923	1.969	up	0.044	Benzene and its derivatives
Tumonoic acid A	1.873	2.6538	up	0.008	Carboxylic acids and derivatives

VIP, variable importance for project; FC, fold change.

**TABLE 5:** Unadjusted and adjusted multivariate logistic regression analysis of metabolites associated with hearing recovery.

Metabolites	Unadjusted		Adjusted	
	Or (95% CI)	*p*-value	Or (95% CI)	*p*-value
N4-Acetylcytidine	3.025 (1.492–7.098)	0.021	3.874 (1.375–8.514)	0.030
p-Phenylenediamine	1.834 (1.073–3.109)	0.042	1.643 (0.854–2.853)	0.118
Sphingosine	3.652 (1.486–8.951)	0.003	2.977 (1.512–7.755)	0.009
Prostaglandin A1	1.279 (0.699–2.708)	0.813	1.314 (0.707–2.892)	0.684
Glycero-3-phosphocholine	2.094 (0.873–4.916)	0.308	1.836 (0.903–5.045)	0.145
Propenoic acid	1.596 (1.128–5.217)	0.038	1.476 (0.915–5.735)	0.208
1,3-Dimethylbenzene	1.902 (0.592–4.083)	0.580	1.767 (0.613–4.982)	0.580
Nonadecanoic acid	2.087 (1.312–7.659)	0.026	1.894 (1.276–6.942)	0.033
2,3,4-Trichlorobiphenyl	1.093 (0.871–3.129)	0.742	1.387 (0.901–2.896)	0.589
Tumonoic acid A	1.472 (0.762–3.238)	0.519	1.089 (0.884–2.676)	0.479

OR, odds rate; CI, confidence interval. Adjusted for age, gender, BMI, atopy, side, tinnitus and vertigo.

## Discussion

To the best of our knowledge, this study was the first one to conduct a novel application of metabolomics to explore serum metabolic profiles of SSNHL patients and identify potential biomarkers for predicting hearing recovery based on distinctive metabolites. Our results demonstrated that SSNHL patients presented discriminative serum metabolites and metabolic pathways compared to HCs, and several metabolites exhibited potential diagnostic values for SSNHL. We also found that SSNHL patients with good hearing recovery manifested significantly different metabolic profiles in comparison with those with poor hearing recovery, and several metabolites were proved to be associated with the auditory recovery and exhibit powerful abilities for predicting the hearing prognosis. Collectively, these results suggested that serum metabolomics was a useful and easily performed method for diagnosing SSNHL and predicting hearing recovery after treatment, which provided a new perspective to understand the underlying mechanism of SSNHL and better personalize its therapies.

Metabolomics is a promising and powerful method for describing the metabolic variety and discovering potential biomarkers for disease phenotype and prognosis prediction (Huang et al., 2022c; Ruan et al., 2022). Recently, the role of metabolic network regulation in inner ear diseases attached close attention and became a new entry point for clarifying the inner ear pathology (Fujita et al., 2015; Trinh et al., 2019). Most previous studies focused on the metabolic changes in the inner ear fluid of hearing loss patients and animal models, and few were known in the peripheral blood. He et al. (2017) found that arginine, proline, and purine metabolic pathways were disturbed in the acoustic trauma rat models in comparison with controls. Trinh and others conducted a metabolomic profile of human perilymph from cochlear-implanted patients and found that metabolic signatures were significantly changed in the duration of hearing loss (Trinh et al., 2019). However, there were few studies investigating the metabolic signatures in the serum of SSNHL patients, and no previous study employed metabolomics to predict hearing recovery.

In the present study, we first observed that fatty acid metabolism was significantly perturbed in the serum of SSNHL patients and involved in the hearing prognosis. Substantial evidence suggested that the metabolism of the fatty acids played a crucial role in maintaining the inner ear and vestibule cellular mechanisms and were involved in hearing and balance system development (Fujita et al., 2015; Pirttilä et al., 2019). Moreover, the inner ear was a high energy consumption organ whose demands on energy supply were enormous (Chen et al., 2022c). When glucose metabolism cannot meet the energy supply in the inner ear under physiological or pathological conditions, fatty acid oxidation will be enhanced (Chen et al., 2022d; Wang et al., 2022). Previous studies suggested that fatty acid metabolism was disturbed when inner hair cells exposing to pathophysiological stimuli; cumulated fatty acids could facilitate oxidative stress injury and apoptosis in the inner hair cells, resulting in acute hearing loss (Fujita et al., 2015; Pirttilä et al., 2019; He et al., 2022; Peter et al., 2022). Here, we observed that several fatty acids metabolic products, including octanoylcarnitine, were changed in the serum of SSNHL, and prostaglandin A1and nonadecanoic acid levels were associated with hearing recovery, suggesting that fatty acids metabolism pathway might contribute to the development of SSNHL and its hearing prognosis. However, the underlying mechanisms were not unknown and required further exploration.

Another interesting finding was that the serum concentrations of sphingosine were increased in the SSNHL patients, and serum sphingosine exhibited prognostic value as a reliable biomarker for predicting hearing recovery before the onset of treatment. Sphingosine, an important member of sphingolipids, is a ubiquitous component of the cell membrane and serves a pivotal role in cell growth, metabolism regulation, signal transduction, and various physiological function (Gaggini et al., 2022; Grewe et al., 2022). Previous publications demonstrated that sphingosine and sphingosine-1-phosphate (its downstream product) were bioactive biomolecules which exerted proinflammatory effects and promoted proapoptotic properties in the vascular endothelial cell, contributing to the occurrence and development of cardiovascular diseases (Bernacchioni et al., 2022; Kurano et al., 2022). In recent studies, sphingosine levels were proven to be shifted in the inner tissues and perilymph and aggravated the oxidative stress and inflammation in sensorineural hearing loss and ototoxicity (Romero-Guevara et al., 2015; Cencetti et al., 2019; Wang et al., 2020; Ramkumar et al., 2021). Ingham et al. (2016) found that sphingolipids metabolism was closely associated with auditory function in progressive hearing loss patients and endocochlear potential decline in mouse models. Collectively, these studies provide a reasonable explanation for our observation of increased sphingosine levels in the serum of SSNHL patients and its predictive value in hearing recovery.

Several limitations exist in the present study, which may affect the reliability of the reported results. First, this work is a preliminary and exploratory study with a relatively small sample size, and the sample size is not a detailed calculation. We conducted multiple data processing analyses and multivariate analyses to recheck the reliability of the results. Second, all subjects were recruited in a single medical centre, which may limit its generation. Third, we do not validate the diagnostic and predictive values of identified metabolites in another validation cohort. Lastly, and no consensus currently exists regarding the hearing recovery evaluation criterion in SSNHL, this may weaken the reliability of the conclusion. Further prospective multicenter studies with a large sample size to further explore SSNHL disease-specific metabolites in different subgroups based on audiogram types and assess their predictive values in hearing recovery in each subgroup.

In conclusion, our study performed the metabolic profiles and indicated that metabolomics could be successfully performed to detect SSNHL-specific metabolic shifts. Our study also identified several metabolites that exhibited potential diagnostic and predictive values in SSNHL, which might contribute to illustrating the mechanisms of SSNHL and provide new insights for its treatments.

## Data Availability

The original contributions presented in the study are included in the article/supplementary material, further inquiries can be directed to the corresponding author.
